# Analysis of the efficacy and safety of inpatient and outpatient initiation of KD for the treatment of pediatric refractory epilepsy using generalized estimating equations

**DOI:** 10.3389/fneur.2023.1146349

**Published:** 2023-04-27

**Authors:** Wei Li, Xiaoyan Hao, Wei Gu, Chao Liang, Fulai Tu, Le Ding, Xiaopeng Lu, Jianxiang Liao, Hu Guo, Guo Zheng, Chunfeng Wu

**Affiliations:** ^1^Department of Quality Management, Children's Hospital of Nanjing Medical University, Nanjing, Jiangsu, China; ^2^Department of Neurology, Children's Hospital of Nanjing Medical University, Nanjing, Jiangsu, China; ^3^Key Laboratory of Environmental Medicine Engineering, Department of Epidemiology and Health Statistics, School of Public Health, Southeast University, Nanjing, Jiangsu, China; ^4^Department of Neurology, Shenzhen Children’s Hospital, Shenzhen, Guangdong, China

**Keywords:** refractory epilepsy, ketogenic diet, pediatric, generalized estimating equations, inpatient initiation, outpatient initiation

## Abstract

**Objective:**

To compare the efficacy and safety of inpatient and outpatient initiation ketogenic diet (KD) protocol of pediatric refractory epilepsy.

**Methods:**

Eligible children with refractory epilepsy were randomly assigned to receive KD with inpatient and outpatient initiation. The generalized estimation equation (GEE) model was used to analyze the longitudinal variables of seizure reduction, ketone body, weight, height, body mass index (BMI), and BMI Z-score at different follow-up times between the two groups.

**Results:**

Between January 2013 and December 2021, 78 and 112 patients were assigned to outpatient and inpatient KD initiation groups, respectively. There were no statistical differences between the two groups based on baseline demographics and clinical characteristics (all *P*s > 0.05). The GEE model indicated that the rate of reduction of seizures≥50% in the outpatient initiation group was higher than that of the inpatient initiation group (*p* = 0.049). A negative correlation was observed between the seizure reduction and blood ketone body at 1, 6, and 12 months (all *Ps* < 0.05). There were no significant differences in height, weight, BMI, and BMI Z-score between the two groups over the 12-month period by the GEE models (all *P*s > 0.05). Adverse events were reported by 31 patients (43.05%) in the outpatient KD initiation group and 46 patients (42.20%) in the inpatient KD initiation group, but these differences were not statistically significant (*p* = 0.909).

**Conclusion:**

Our study shows that outpatient KD initiation is a safe and effective treatment for children with refractory epilepsy.

## Introduction

Children and adolescents with epilepsy are at increased risk for poor long-term intellectual and psychosocial outcomes, along with a poor health-related quality of life (1). Epilepsy accounts for over 13 million disability-adjusted life years (DALYs) and is responsible for more than 0.5% of the global burden of disease (2). Although many underlying disease mechanisms can lead to epilepsy, the cause of the disease is still unknown in about 50% of cases globally (3). Structural, genetic, brain injury, vascular causes, and central nervous system infection are the most frequently identified risk factors (4), head injury, hypoxic–ischemic encephalopathy, and infections of the brain are significant risk factors for childhood epilepsy (5, 6). It is estimated that 49–139 per 100,000 people are diagnosed with epilepsy each year (7) and the point prevalence of active epilepsy was 6.38 per 1,000 population (8). The number of people with epilepsy is expected to increase further.

In China, it is estimated that 10 million people have epilepsy, but only around a third of them receive appropriate or adequate treatment (9). Antiepileptic drugs (AEDs) are the main approach to epilepsy treatment and achieve seizure freedom (10). However, they have not substantially altered the overall seizure-free outcomes, the failure to achieve sustained seizure freedom with adequate uses of two AEDs was defined as refractory epilepsy (11, 12). Currently, several non-pharmacologic interventions can be used to treat refractory epilepsy, such as metabolic therapy, brain stimulation therapy, and complementary therapy (13). The ketogenic diet (KD) is a diet that has been used since the early 1920s to control seizures in patients (14). The KD is very high in fats and extremely low in carbohydrates and protein, which promises to decrease seizure frequency significantly for patients with refractory epilepsy (15). Moreover, compared to AEDs and other treatments, the KD is inexpensive, fairly easy to implement, and the use of KD for anti-epileptics is increasing globally.

The KD is usually initiated during hospitalization, and patients are closely monitored under expert guidance, especially in cases of infants and children with severe co-morbidities (16). The 2019 coronavirus disease (COVID-19) pandemic and the associated lock-down measures have certainly affected children with epilepsy, particularly those who are candidates for KD (17), as a result, inpatient KD initiation has been sharply restricted (18). Therefore, continuing a specific diet during the COVID-19 pandemic is expected to be difficult in some aspects (19). Outpatient KD initiation that will result in easier implementation, shorter hospital stays, and reduced medical and family costs would have clinical and economic advantages (20). Some epilepsy centers are now beginning the KD by using an outpatient initiation protocol, however, this is not a general routine in many centers, and most KD centers still follow an inpatient protocol (21). There was no evidence to prove that inpatient initiation of the ketogenic diet was superior to outpatient initiation concerning long-term seizure control or cognitive improvement (22). This study aimed to compare the efficacy and safety of inpatient and outpatient initiation KD protocol in children with refractory epilepsy and to evaluate the differences in blood ketone body, weight, height, and BMI in children between the inpatient and outpatient initiation of the KD.

## Materials and methods

### Study design and participants

**Figure 1:**
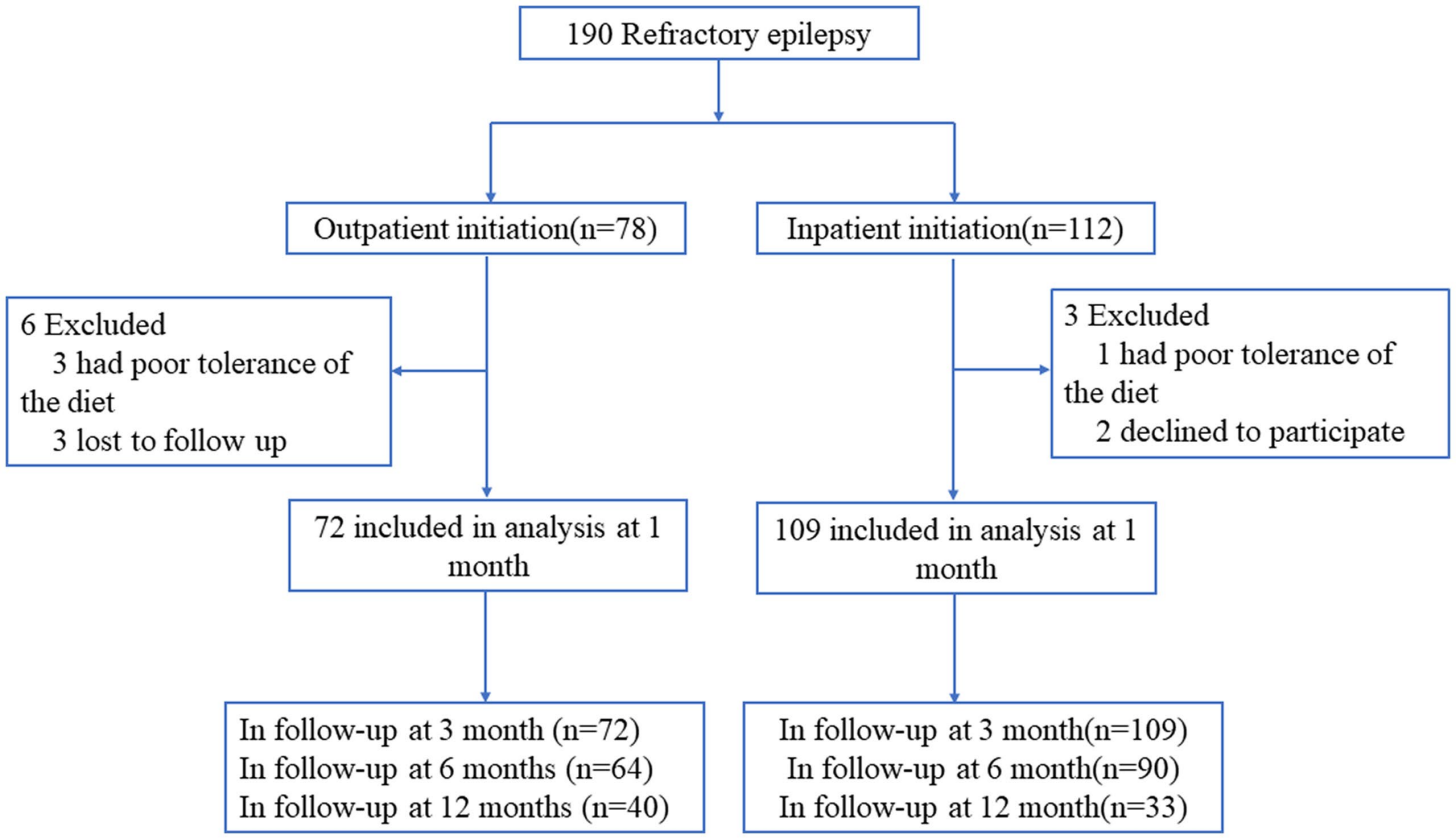
Detail of participants’ flow.

From January 2013 to December 2021, children with refractory epilepsy in the epilepsy center of Children’s Hospital of Nanjing Medical University were enrolled and underwent a ketogenic diet program. Inclusion criteria: the patients ≤16 years old; the patients have tried at least two AEDs; the patients have at least three countable seizures per week; the parents are willing to include their children in the study after written and verbal information; and they were motivated to and capable of adhering to the ketogenic diet. Exclusion criteria: previous treatment with the KD; the patient’s seizures are under acceptable control; with known or suspected inborn errors of metabolism in which KD is contraindicated; changes in the AED type or dose in 1 month before the inclusion; participating in any other epilepsy-related study. We used computer-generated random numbers to randomize patients to receive KD treatment by inpatient initiation or outpatient initiation (in a ratio of 1.5:1). Of 190 children with refractory epilepsy, 112 patients were assigned to the inpatient KD initiation group, and 78 patients were assigned to the outpatient KD initiation group. Detail of participants’ flow including all study exclusions and withdrawals are shown in Figure 1.

### KD procedures

Our KD management group consists of physicians, nutritionists, and nurses. A gradual-initiation, non-fasting classic KD treatment protocol was used, which was started at a 1:1 ratio (lipids: nonlipids). Lipid content was gradually increased to 2:1, 2.5:1, 3:1, and 4:1 (23).

Inpatient KD initiation was admitted inpatients and closely monitored for the internal environment and any acute adverse effects during the first week in the hospital. Outpatient KD initiation included 1-day case admission for baseline measurements and parents were educated. All patients were monitored by the medical team regularly to look out for side effects, to ensure nutritional needs are being met, and to assess the diet’s effect on seizure control.

After admission, the KD management group educated the children’s families to prepare the KD at home depending on the child’s regular waking and eating schedule. The data on the children’s seizures and diet (seizure time, manifestation, frequency, duration, KD conditions, and adverse reactions) were recorded by their families. The length of KD treatment was 12 months.

### Outcome

The primary clinical outcome was a ≥ 50% reduction in seizure frequency (24) after the introduction of KD. The percentage of patients achieving ≥50% reduction in seizure frequency was assessed at 1, 3, 6, and 12 months of KD treatment.

Body weight was measured to the nearest 0.1 kg using a calibrated electronic scale with participants in light clothing and without shoes. Height was measured to the nearest 0.1 cm with participants without shoes. BMI was computed as kg/m^2^. Blood ketone body concentrations were measured using an electrochemical capillary blood monitor device with the corresponding individually foil-wrapped test strips for Beta-hydroxybutyrate (BHB).

### Adverse events

Adverse effects were evaluated using parental questionnaires, including diarrhea, vomiting, anorexia, constipation, decrease in immunity, slow growth, and so on. Participants responded with “yes” or “no,” depending on the presence of symptoms. All adverse effects were listed in Supplementary Table S1.

### Statistical analysis

R version 4.1.0 was used for data cleaning and statistical analyses. Data are presented as mean ± standard deviation or frequency (%) as appropriate. The variables between the two groups were compared using independent samples *t* and Pearson’s Chi-square tests. The generalized estimation equation (GEE) model was used to analyze the longitudinal variables of seizure reduction, ketone body, weight, height, BMI, and BMI Z-score at different follow-up times. The significance level was considered when *p* < 0.05.

### Ethics approval

This study was reviewed and approved by the Human Research Ethics Committee of the Children’s Hospital of Nanjing Medical University (approval ID: 201312030). The patients were recruited during a multi-center study initiated by Jianxiang Liao’s team at Shenzhen Children’s Hospital (Clinical trial registration: ChiCTR-IIR-16008342). All participating subjects received written detailed information and signed consent forms for the interview and the processing of sensitive personal data by their parents. The procedure of our study was performed following the principles stated in the Declaration of Helsinki.

## Results

### The demographic and clinical characteristics at baseline

Between January 2013 and December 2021, a total of 190 patients were included in the study, among them, 78 patients were assigned to the outpatient KD initiation group and 112 patients were assigned to the inpatient KD initiation group. Nine patients withdrew before 1 month follow-up, six from the outpatient initiation group and three patients from the inpatient initiation group. Thus leaving 181 patients for intention-to-treat analysis including 109 in the inpatient initiation group and 72 in the outpatient initiation group. Of the 181 patients who remained in the study groups, 116 were males and 65 were females, and 114 patients were younger than 3 years old. Baseline seizure frequency was more than 5 times/day in 123 children, 50.83% were on three or more AEDs, and spasms was the main seizure type (59.67%, 108/181), followed by tonic (20.44%, 37/181), multiple (9.94%, 18/181), and other (9.94%, 18/181).The average blood ketone body, weight, height, BMI, and BMI Z-score at KD initiation of the study groups were 2.47 ± 0.87 mmol/L, 14.20 ± 5.90 kg, 92.32 ± 17.97 cm, 16.16 ± 2.26 kg/m^2^, and 0.15 ± 1.56, respectively (Table 1). Baseline demographics and clinical characteristics were comparable between the two groups (Table 1). There were no statistical differences between the two groups based on gender, age seizure type, duration of seizure onset, number of AEDs, seizure frequency, type of seizure, weight, height, BMI, and BMI Z-score at KD initiation (all *Ps* > 0.05). The proportion of patients completing ≥12 months of treatment was higher in the outpatient KD initiation group [40 (55.56%)] than in patients in the inpatient initiation [33 (30.28%); *p* < 0.001].

**Table 1:** Demographics and clinical characteristics at baseline.

Variables		Total (*n* = 181)	Outpatient (*n* = 72)	Inpatient (*n* = 109)	$x^{2}$ /*t*	*p* value
Gender	Male	116 (64.09%)	49 (68.06%)	67 (61.47%)	0.82	0.366
	Female	65 (35.91%)	23 (31.94%)	42 (38.53%)		
Age at KD initiation	<3 years	114 (62.98%)	41 (56.94%)	73 (66.97%)	2.35	0.309
	3–5 years	46 (25.41%)	20 (27.78%)	26 (23.85%)		
	≥6	21 (11.60%)	11 (15.28%)	10 (9.17%)		
Duration of seizure onset at KD initiation	<2 years	126 (62.50%)	45 (62.50%)	81 (74.31%)	2.86	0.091
	≥2 years	55 (37.50%)	27 (37.50%)	28 (25.69%)		
Number of AEDs at initiation of KD	2	89 (49.17%)	36 (50.00%)	53 (48.62%)	0.03	0.856
	>2	92 (50.83%)	36 (50.00%)	56 (51.38%)		
Seizure frequency	≤5/day	78 (43.09%)	28 (38.89%)	50 (45.87%)	0.86	0.355
	>5/day	103 (56.91%)	44 (61.11%)	59 (54.13%)		
Type of seizure	Spasms	108 (59.67%)	47 (65.28%)	61 (55.96%)	2.71	0.439
	Tonic	37 (20.44%)	12 (16.67%)	25 (22.94%)		
	Multiple	18 (9.94%)	5 (6.94%)	13 (11.93%)		
	Other	18 (9.94%)	8 (11.11%)	10 (9.17%)		
Ketone body at KD initiation (mmol/L; mean ± SD)		2.47 ± 0.87	2.34 ± 0.76	2.55 ± 0.87	−1.58	0.116
Weight at KD initiation (kg; mean ± SD)		14.20 ± 5.90	14.88 ± 6.05	13.75 ± 5.77	1.26	0.210
Height at KD initiation (cm; mean ± SD)		92.32 ± 17.97	95.41 ± 18.42	90.29 ± 17.46	1.89	0.060
BMI at KD initiation (kg/m^2^; mean ± SD)		16.16 ± 2.26	15.86 ± 2.48	16.36 ± 2.08	−1.49	0.139
BMI Z-score at KD initiation (mean ± SD)		0.15 ± 1.56	0.03 ± 1.82	0.23 ± 1.36	−0.82	0.414

### Comparison of the efficacy in the different groups and follow-up times based on the GEE model

In patients completing 1-month (*n* = 181), 3 months (*n* = 181), 6 months (*n* = 152), and 12 months (*n* = 72) of KD treatment, the rate of seizure reduction ≥50% was 25.41, 43.09, 49.35, and 65.75%, respectively. For patients in the outpatient initiation group, the rate of seizure reduction ≥50% was 31.94, 51.39, 56.25, and 69.70%, at 1, 3, 6, and 12 months, respectively. The GEE model indicated that the rate of seizure reduction ≥50% in the outpatient initiation group was higher than that of the inpatient initiation group (*p* = 0.049). Compared with 12 months, the rate of seizure reduction ≥50% was lower at 1, 3, and 6 months (all *P*s < 0.01; Table 2).

**Table 2:** Comparison of the efficacy in the different groups and follow up times based on the GEE.

Groups	1 month		3 months		6 months		12 months		*β* (*95%CI*)	*p*
	<50%	≥50%	<50%	≥50%	<50%	≥50%	<50%	≥50%		
Total	135 (74.59%)	46 (25.41%)	103 (56.91%)	78 (43.09%)	78 (50.65%)	76 (49.35%)	25 (34.25%)	48 (65.75%)		
Inpatient initiation	86 (78.90%)	23 (21.10%)	68 (62.39%)	41 (37.61%)	50 (55.56%)	40 (44.44%)	15 (37.50%)	25 (62.50%)	Ref	
Outpatient initiation	49 (68.06%)	23 (31.94%)	35 (48.61%)	37 (51.39%)	28 (43.75%)	36 (56.25%)	10 (30.30%)	23 (69.70%)	0.51 (0.00,1.10)	0.049
*β* (*95%CI*)	−1.73 (−2.27, −1.18)		−0.92 (−1.42, −0.41)		−0.67 (−1.14, −0.20)		Ref			
*p value*	<0.001		<0.001		0.005					

### Comparison of the blood ketone body in the different groups and follow up times based on the GEE

No differences were observed at the beginning of KD initiation between inpatient and outpatient groups for blood ketone body concentration (Table 1). The GEE model indicated that after 12 months of KD treatment, blood ketone body concentrations of patients were higher in the inpatient initiation group than in the outpatient initiation group (*p* = 0.066). Compared with the baseline, there was no significant difference in blood ketone body concentrations at 1, 3, 6, and 12 months (all *Ps* > 0.05; Table 3).

**Table 3:** Comparison of the blood ketone body in the different groups and follow-up times based on the GEE.

Groups	Baseline	1 month	3 months	6 months	12 months	*β (95%CI)*	*P-value*
Total	2.47 ± 0.89	2.58 ± 0.78	2.55 ± 0.87	2.52 ± 0.78	2.34 ± 0.80		
Inpatient initiation	2.55 ± 0.87	2.73 ± 0.82	2.58 ± 0.88	2.52 ± 0.77	2.36 ± 0.77	Ref	
Outpatient initiation	2.34 ± 0.86	2.35 ± 0.65	2.50 ± 0.85	2.51 ± 0.78	2.33 ± 0.84	−0.16(−0.33, 0.01)	0.066
*β (95%CI)*	Ref	0.11 (−0.01, 0.23)	0.09 (−0.06, 0.23)	0.05 (−0.11, 0.22)	−0.11 (−0.32, 0.09)		
*P-value*		0.064	0.254	0.516	0.282		

### The relation between ketone body and the rate of seizure reduction ≥50%

It is apparent from Table 4 that there is a relation between the rate of seizure reduction ≥50% and blood ketone body level. Spearman’s rank correlation established a negative correlation between the rate of seizure reduction ≥50% and blood ketone body level at 1, 6, and 12 months (all *P*s < 0.05).

**Table 4:** The relation between ketone body and the rate of seizure reduction ≥50% at different follow up time.

Ketone body	The rate of seizure reduction ≥50%			
	1 month (*n* = 181)	3 months (*n* = 181)	6 months (*n* = 154)	12 months (*n* = 73)
r	−0.17	−0.13	−0.24	−0.24
*P*	0.022	0.088	0.002	0.041

### Comparison of the impact of the KD on growth in children between inpatient and outpatient KD initiation groups

**Figure 2:**
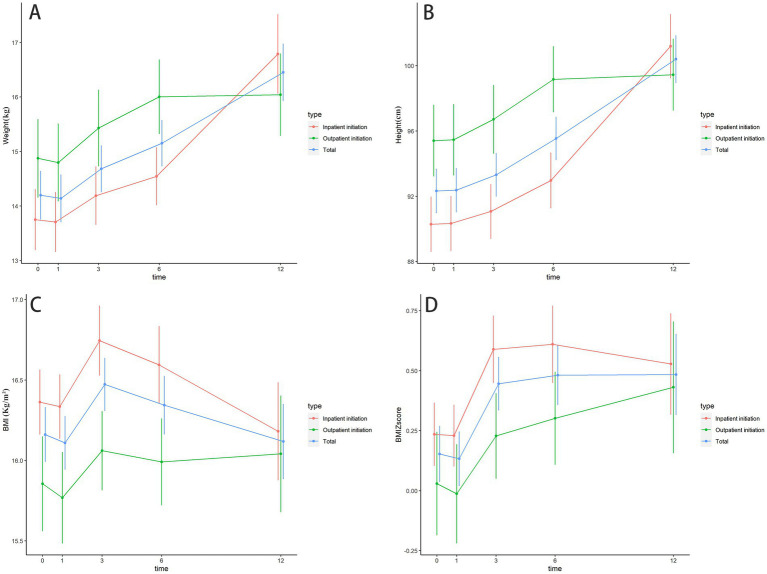
The changes in the growth of patients at different follow up times. **(A)** The changes in the weight of patients at different follow up times; **(B)** The changes in height of patients at different follow up times; **(C)** The changes in BMI of patients at different follow up times; **(D)** The changes in BMI Z-score of patients at different follow up times.

Baseline weight, height, BMI, and BMI Z-score were not significantly different between different KD initiation groups (Table 1), the changes in the growth of patients at different follow-up times were shown in Supplementary Table S2 and Figure 2. Table 5 shows the results of the GEE model, there were no significant differences in height, weight, BMI, and BMI Z-score between the inpatient KD initiation group and outpatient KD initiation group over the 12-month period by the GEE models (all *P*s > 0.05). The GEE models also revealed that the mean weight was significantly higher at 3, 6, and 12 months than at the baseline (all *P*s < 0.001). Compared to the baseline, the mean height was significantly higher at 1, 3, 6, and 12 months (all *P*s < 0.05). For BMI, a significant increase in BMI was observed at the second follow-up (3 months) than the baseline (*p* = 0.002). BMI-z score was significantly increased after the intervention at 3, 6, and 12 months than the baseline (all *P*s < 0.05).

**Table 5:** Comparison of the growth in the different groups and follow-up times based on the GEE.

Variables		Weight		Height		BMI		BMI Z-score	
		*β* (95%CI)	*p* value	*β* (95%CI)	*p* value	*β* (95%CI)	*p* value	*β* (95%CI)	*p* value
Type of KD initiation	Inpatient initiation	Ref		Ref		Ref		Ref	
	Outpatient initiation	1.03 (−0.58, 2.64)	0.210	4.80 (−0.09, 9.69)	0.055	−0.55 (−1.15, 0.06)	0.077	−0.26 (−0.69, 0.17)	0.230
Time	Baseline	Ref		Ref		Ref		Ref	
	1 month	−0.06 (−0.15, 0.03)	0.217	0.05 (0.01, 0.09)	0.024	−0.05 (−0.15, 0.05)	0.319	−0.02 (−0.09, 0.05)	0.586
	3 months	0.49 (0.35, 0.62)	<0.001	0.99 (0.73, 1.25)	<0.001	0.31 (0.12, 0.51)	0.002	0.29 (0.15, 0.43)	<0.001
	6 months	0.94 (0.45, 1.42)	<0.001	3.14 (1.68, 4.60)	<0.001	0.19 (−0.08, 0.47)	0.174	0.33 (0.13, 0.53)	0.001
	12 months	2.20 (1.20, 3.20)	<0.001	7.83 (4.92, 10.73)	<0.001	−0.01 (−0.45, 0.42)	0.952	0.35 (0.03, 0.66)	0.029

### Adverse effects

Adverse events were reported by 31 patients (43.05%) in the outpatient KD initiation group, and 46 patients (42.20%) in the inpatient KD initiation group, and no statistical differences (*p* = 0.909) in the incidence of AE were found between the two groups (Table 6). Serious adverse events were not reported in the two groups. The most common adverse events were diarrhea [outpatient group, 6 (19.35%); inpatient group, 9 (20.93%)], anorexia [outpatient group, 7 (22.58%); inpatient group, 8 (18.60%)], constipation [outpatient group, 3 (9.68%); inpatient group, 8 (18.60%)], slow growth [outpatient group, 4 (12.90%); inpatient group, 5 (11.63%)], and sleep disorder [outpatient group, 4 (12.90%); inpatient group, 4 (4.65%); Table 6].

**Table 6:** The distribution of adverse effects.

Adverse effects	Outpatient		Inpatient		Total	
	Frequency	Composition ratio	Frequency	Composition ratio	Frequency	Composition ratio
Diarrhea	6	19.35%	9	20.93%	15	20.00%
Anorexia	7	22.58%	8	18.60%	15	20.00%
Constipation	3	9.68%	8	18.60%	11	14.67%
Slow growth	4	12.90%	5	11.63%	9	12.00%
Sleep disorder	4	12.90%	2	4.65%	6	8.00%
Decrease in immunity	4	12.90%	0	0.00%	4	5.33%
Vomit	0	0.00%	4	9.30%	4	5.33%
Dyslipidemia	2	6.45%	2	4.65%	4	5.33%
Increased seizures	1	3.23%	1	2.33%	3	4.00%
Hypoglycemia	0	0.00%	1	2.33%	1	1.33%
Allergy	0	0.00%	1	2.33%	1	1.33%
Myocardial enzyme abnormalities	0	0.00%	1	2.33%	1	1.33%
Transaminase abnormalities	0	0.00%	1	2.33%	1	1.33%

## Discussion

Ketogenic diet is an ideal therapeutic tool for people with epilepsy and in particular for children with refractory epilepsy (25). More than 80% of the centers still routinely begin the classic KD in the hospital, to better follow the children, promptly operate the necessary adjustments, and train the parents to be able to properly manage epilepsy at home (16). Initiation of the KD in an inpatient setting is costly and often involves a considerable social and economic burden for parents and children, which caused barriers for patients to gain access to the KD (14). Since the outbreak of COVID-19 in early 2020, people’s life, study, work, and medical treatment were seriously affected (26). Due to the measure of city-wide lockdowns, hospital admissions fell dramatically (27), people with epilepsy and their caregivers reported an overall increase in seizures, with difficulties accessing medical care, particularly medications, investigations, information, and self-management (28). Thus, the inpatient initiation of KD for epilepsy has been severely impacted, highlighting the importance of outpatient initiation of KD.

Previous studies suggested that outpatient KD initiation is no worse than inpatient initiation in terms of effectiveness (16, 29), a significantly better response was observed in the outpatient KD initiation group in the treatment of refractory epilepsy in our study. On the other hand, we found the 12-month retention rate of the outpatient KD initiation group was higher than that of the inpatient KD initiation group. Moreover, there was no statistical difference in the comparison results in terms of blood ketone value, growth, and side effects. Our findings support the initiation of a ketogenic diet in outpatient settings. Telemedicine use among healthcare providers and patients was increased because of the COVID-19 pandemic (28, 30), and it has been helping healthcare workers to assess, diagnose, monitor, treat, and educate patients. With the help of telemedicine, we can greatly promote the popularization of outpatient initiation of KD, and facilitate the two-way interaction between patients and the management team of the ketogenic diet.

While consuming a ketogenic diet, ketone bodies were generated in the mitochondrial matrix of liver cells (31), which have long been thought to have a direct antiseizure effect but have not been proven (32). Ketone bodies include BHB, acetoacetic acid, and acetone. For clinical purposes, blood BHB testing is widely used for assessing ketone bodies (33). Previous studies have found inconsistent results on the relationship between blood BHB and seizure control (32, 34–39). In our study, we found that the blood BHB was increased at the beginning of KD initiation consistence with a similar previous study (34). We also observed a negative correlation between the blood ketone body and the reduction in seizure frequency at 1, 6, and 12 months, however, the ultimate validation of their role in the clinical setting has yet to be firmly demonstrated (38).

A KD is known to lead weight loss and is considered to be metabolically healthy (40). Several reports have indicated poor linear growth in children and very young children grow poorly on the diet (41, 42). However, our findings were opposite to existing literature, and a growth deficit was identified in only 5% (9/181) of the study participants, this ratio is significantly lower than previous study (43, 44). The sample size of our study is larger than similar studies (43, 44), so our findings are accurate and representative. Taken together, our findings demonstrate that growth retardation may occur in a minority of patients treated with KD.

Our data indicated adverse effects of the KD were mild, vomiting, constipation, and diarrhea were reported commonly. Because a gradual-initiation KD was used in our study, lipid content was gradually increased, this gradual process can avoid metabolic acidosis (45), and give children enough time to tolerate the ketogenic diet, resulting in fewer adverse effects (46). Although it was not sure whether these adverse effects are actually due to the implemented KD or perhaps an underlying disorder, KD is required to be performed under careful medical supervision. The family should also be informed about how to recognize the symptoms of adverse effects early. Most of the side effects were tolerable after adjustment of the KD and symptomatic treatment (47).

## Conclusion

Our study shows that outpatient KD initiation is a safe and effective treatment for children with refractory epilepsy. There was no statistical difference in the comparison results in terms of blood ketone body, growth, and side effects between different initiation of KD, and therefore, outpatient initiation of KD should be encouraged.

## Limitations

Our study also has some limitations that need to be addressed. First, the sample size is relatively small, and the simple randomization may have resulted in an unequal number of participants among groups. However, we compared the baseline data of the two groups, and there was no statistical difference. Second, we did not conduct an adherence survey of those patients who discontinued ketogenic diet intervention during the follow-up period. Third, the missing data in the follow-up coul have biased our results, even though we used the GEE model to handle missing values in longitudinal data.

## Data availability statement

The raw data supporting the conclusions of this article will be made available by the authors, without undue reservation.

## Ethics statement

This study was reviewed and approved by the Human Research Ethics Committee of the Children’s Hospital of Nanjing Medical University (approval ID: 201312030). Written informed consent to participate in this study was provided by the participants’ legal guardian/next of kin.

## Author contributions

WL: writing-original draft. XH: methodology. WG: formal analysis. CL: investigation. FT: software. LD: resources. XL: data curation. JL: supervision. HG: project administration. GZ: writing-review and editing. CW: conceptualization and funding acquisition. All authors contributed to the article and approved the submitted version.

## Funding

The study was financed by the Medical Scientific Research Project of Jiangsu Provincial Health Commission (ZD2022053) and China Postdoctoral Science Foundation (2022M721682).

## Conflict of interest

The authors declare that the research was conducted in the absence of any commercial or financial relationships that could be construed as a potential conflict of interest.

## Publisher’s note

All claims expressed in this article are solely those of the authors and do not necessarily represent those of their affiliated organizations, or those of the publisher, the editors and the reviewers. Any product that may be evaluated in this article, or claim that may be made by its manufacturer, is not guaranteed or endorsed by the publisher.

## Supplementary material

The Supplementary material for this article can be found online at: https://www.frontiersin.org/articles/10.3389/fneur.2023.1146349/full#supplementary-material
