# Supplementary material for: Analysis of the efficacy and safety of inpatient and outpatient initiation of KD for the treatment of pediatric refractory epilepsy using generalized estimating equations
Source: Front Neurol. 2023 Apr 27;14:1146349. doi: 10.3389/fneur.2023.1146349 (PMC10174452; doi:10.3389/fneur.2023.1146349)
Supplement: Supplementary file 1 [file Table_1.DOCX]

Table S1 the list of adverse effect

| ID | Adverse effect |
| --- | --- |
| 1 | Diarrhea |
| 2 | Anorexia |
| 3 | Constipate |
| 4 | Slow growth |
| 5 | Sleep disorder |
| 6 | Weak in immunity |
| 7 | Vomit |
| 8 | Dyslipidemia |
| 9 | Increased seizures |
| 11 | Hypoglycemia |
|  | Hypoproteinemia |
| 12 | Allergy |
| 13 | Myocardial enzyme abnormalities |
| 14 | Transaminase abnormalities |
| 15 | Infection disease |
| 16 | Fever |
| 17 | Hypertriglyceridemia |
| 18 | Acidosis |
| 19 | Lack of energy/fatigue |
| 20 | kidney stones |
| 21 | Others |

Table S2 the changes in growth at different follow up time

|  |  | Baseline | 1 month | 3 months | 6 months | 12 months |
| --- | --- | --- | --- | --- | --- | --- |
| Weight | Total (kg; mean ± SD) | 14.20±5.90 | 14.14±5.79 | 14.68±5.70 | 15.15±5.21 | 16.45±4.44 |
|  | Inpatient (kg; mean ± SD) | 13.75±5.77 | 13.71±5.64 | 14.19±5.53 | 14.55±4.95 | 16.79±4.57 |
|  | Outpatient (kg; mean ± SD) | 14.88±6.05 | 14.80±5.98 | 15.43±5.91 | 16.00±5.43 | 16.04±4.31 |
| Height | Total (cm; mean ± SD) | 92.32±17.97 | 92.37±17.94 | 93.31±17.63 | 95.55±16.18 | 100.41±12.34 |
|  | Inpatient (cm; mean ± SD) | 90.29±17.46 | 90.33±17.44 | 91.07±17.31 | 92.97±15.93 | 101.20±12.29 |
|  | Outpatient (cm; mean ± SD) | 95.41±18.42 | 95.47±18.37 | 96.72±17.70 | 99.17±15.95 | 99.45±12.51 |
| BMI | Total (kg/m^2^; mean ± SD) | 16.16±2.26 | 16.11±2.21 | 16.47±2.20 | 16.34±2.23 | 16.12±1.97 |
|  | Inpatient (kg/m^2^; mean ± SD) | 16.36±2.08 | 16.34±2.07 | 16.75±2.24 | 16.59±2.27 | 16.18±1.91 |
|  | Outpatient (kg/m^2^; mean ± SD) | 15.86±2.48 | 15.77±2.39 | 16.06±2.07 | 15.99±2.15 | 16.04±2.07 |
| BMI Z-score | Total (mean ± SD) | 0.15±1.56 | 0.13±1.51 | 0.45±1.47 | 0.48±1.53 | 0.48±1.43 |
|  | inpatient (mean ± SD) | 0.23±1.36 | 0.23±1.33 | 0.59±1.45 | 0.61±1.51 | 0.53±1.33 |
|  | outpatient (mean ± SD) | 0.03±1.82 | -0.01±1.74 | 0.23±1.50 | 0.30±1.54 | 0.43±1.57 |
